# Localized Myocardial Anti-Inflammatory Effects of Temperature-Sensitive Budesonide Nanoparticles during Radiofrequency Catheter Ablation

**DOI:** 10.34133/2022/9816234

**Published:** 2022-05-31

**Authors:** Ye Liu, Lingling Xu, Qiuyun Zhang, Yong Kang, Lifeng Liu, Zheng Liu, Yuxing Wang, Xuejiao Jiang, Yizhu Shan, Ruizeng Luo, Xi Cui, Yuan Yang, Xinchun Yang, Xiaoqing Liu, Zhou Li

**Affiliations:** ^1^Heart Center & Beijing Key Laboratory of Hypertension, Beijing Chaoyang Hospital, Capital Medical University, Beijing 100020, China; ^2^Beijing Key Laboratory of Micro-Nano Energy and Sensor, Beijing Institute of Nanoenergy and Nanosystems, Chinese Academy of Sciences, Beijing 101400, China; ^3^School of Nanoscience and Technology, University of Chinese Academy of Sciences, Beijing 100049, China; ^4^School of Traditional Chinese Medicine, Capital Medical University, Beijing 100069, China; ^5^Academy of Medical Engineering and Translational Medicine, Medical College, Tianjin University, Tianjin 300072, China; ^6^Center on Nanoenergy Research, School of Physical Science and Technology, Guangxi University, Nanning 530004, China; ^7^Institute for Stem Cell and Regeneration, Chinese Academy of Sciences, Beijing 100101, China

## Abstract

Radiofrequency (RF) catheter ablation has emerged as an effective alternative for the treatment of atrial fibrillation (AF), but ablation lesions will result in swelling and hematoma of local surrounding tissue, triggering inflammatory cell infiltration and increased release of inflammatory cytokines. Some studies have shown that the inflammatory response may be related to the early occurrence of AF. The most direct way to inhibit perioperative inflammation is to use anti-inflammatory drugs such as glucocorticoids. Here, we prepared polylactic-co-glycolic acid (PLGA) nanoparticles loaded with budesonide (BUD) and delivered them through irrigation of saline during the onset of ablation. Local high temperature promoted local rupture of PLGA nanoparticles, releasing BUD, and produced a timely and effective local myocardial anti-inflammatory effect, resulting in the reduction of acute hematoma and inflammatory cell infiltration and the enhancement of ablation effect. Nanoparticles would also infiltrate into the local myocardium and gradually release BUD ingredients to produce a continuous anti-inflammatory effect in the next few days. This resulted in a decrease in the level of inflammatory cytokine IL-6 and an increase of anti-inflammatory cytokine IL-10. This study explored an extraordinary drug delivery strategy to reduce ablation-related inflammation, which may prevent early recurrence of AF.

## 1. Introduction

Atrial fibrillation (AF) is the most common persistent arrhythmia in clinical practice, affecting more than 30 million individuals worldwide and resulting in a substantial medical burden globally [1]. Radiofrequency catheter ablation (RFCA) is considered as first-line treatment for some types of atrial fibrillation because of its superiority for the maintenance of sinus rhythm [2]. Early recurrence was observed in about 45% of patients within the first 3 months after ablation [3]. It can be highly symptomatic and frustrating to both patient and electrophysiologist who perform the procedure. It always requires antiarrhythmic drugs (AADs) for cardioversion. However, AADs have significant side effects [4] and limited efficacy [5]. Therefore, how to reduce the recurrence of postoperative atrial fibrillation is a problem worthy of study.

During ablation, the catheter electrode releases radiofrequency current while irrigating cold saline through pores at the tip. This energy produces a high temperature in the targeted local myocardial tissue causing myocardial tissue denaturation, and death. This myocardial tissue no longer plays the role of transmitting conductive signals. However, local damage to the myocardium will lead to obvious hematoma and inflammation of the tissues that surrounds the ablation sites, triggering inflammatory cell infiltration and inflammatory cytokine release. Many studies have shown that the inflammatory response after ablation may be related to the early occurrence of atrial arrhythmia; the level of serum inflammatory markers (IL-6, IL-8, IL-10, TNF-*α*, etc.) is related to the pathogenesis and prognosis of atrial fibrillation [6, 7]. Therefore, ameliorating the inflammatory response after ablation may be a feasible way to reduce the postoperative early recurrence of atrial fibrillation. There are many ways to inhibit the activation of inflammation; the most direct way to reduce perioperative inflammation is to use glucocorticoids [8, 9]. A study showed that intravenous injection of hydrocortisone within three days after ablation and daily oral prednisolone three days after ablation could not only prevent the immediate recurrence of AF but also decrease the recurrence of AF during the midterm follow-up period [10]. However, the systemic application of glucocorticoids must reach a certain threshold in order to reach the ablation site and reduce local inflammation, which may lead to the side effects related to glucocorticoid overuse. Nanotechnology is particularly useful in addressing these challenges [11, 12]. At present, a variety of nanoparticles have been used in the delivery of anti-inflammatory drugs, which can not only improve the stability of drugs but also improve the pharmacokinetics [13–15].

**Figure 1:**
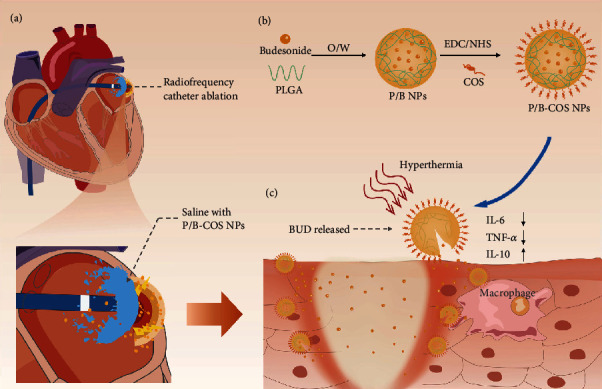
Schematic illustration of P/B-COS NPs delivery during radiofrequency ablation. (a) Radiofrequency ablation with cold saline irrigation, the drugs contained in saline can enter local tissues. (b) Schematic illustration of P/B-COS NPs preparation. NPs were prepared by an oil-in-water emulsion solvent evaporation method and COS was attached to the surface of PLGA NPs by amide bonds. (c) The rapid increased local temperature generated by ablation exceeds the transition temperature of PLGA, resulting in the release of BUD. Nanoparticles infiltrated into local tissue and continued BUD release and anti-inflammatory effect in the following days.

Herein, we propose an extraordinary drug delivery strategy by utilizing the process of RFCA requiring cold saline irrigation. During radiofrequency ablation, glucocorticoid preparation is added to cold saline, and the drug is sprayed directly on the myocardial surface of the ablation site for local administration, to improve the drug content at the ablation site to enhance the local anti-inflammatory effect and reduce the systemic drug dosage and side effects (Figure 1(a)). The drug delivery system we propose possesses the following features: (1) we chose budesonide (BUD), a highly effective local anti-inflammatory drug as the model drug. At present, it is widely used in the treatment of respiratory diseases and inflammatory bowel disease [16], with fewer whole-body side effects [17]. (2) FDA-approved PLGA has been applied to injectable drug subsidiaries due to its biodegradable and biocompatible characteristics [18, 19]. We designed a PLGA nanoparticle internally loaded with BUD (P/B NPs). (3) The nanoparticles prepared by carboxyl end-sealed PLGA are negatively charged. Chitosan oligosaccharide (COS) was crosslinked with PLGA (P-COS NPs) to obtain positively charged nanoparticles, which can better adhere to the negatively charged cell membrane and improve local drug delivery efficiency (Figure 1(b)) [20, 21]. Meanwhile, studies have shown that COS has good immunomodulatory effect, which is expected to play a synergistic anti-inflammatory effect with BUD [22]. (4) The glass transition temperature of PLGA we selected is about 45°C [23]. When the nanoparticles reach the local myocardium, due to the local high temperature generated by resistance heating (≥60°C), PLGA NPs ruptured, and the drug was released into the local myocardium to produce the local anti-inflammatory effect and realize immediate administration response (Figure 1(c)) [24, 25]. PLGA particles will also infiltrate local tissue and continue to release BUD content in the following days. This study explored a new method of BUD solution administration with PLGA as a carrier. The catheter was flushed with saline during ablation; this method minimized the dose of glucocorticoid in ablated local myocardium and enhances the local anti-inflammatory effect.

## 2. Results

### 2.1. Characterization of P/B-COS NPs

**Figure 2:**
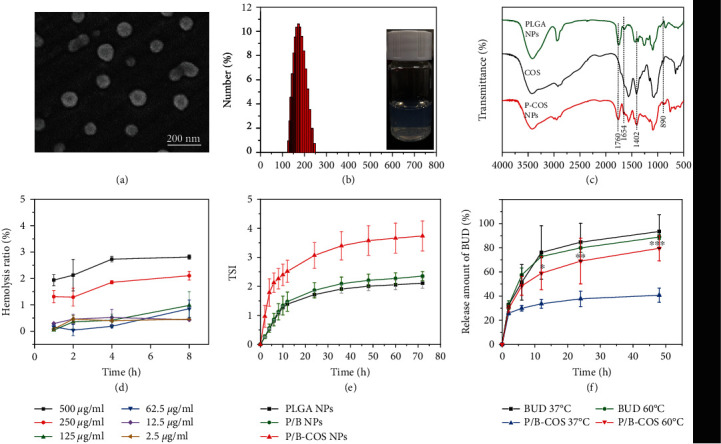
Characterization of NPs. (a) Scanning electron microscope (SEM) image of P/B-COS NPs. (b) Dynamic light scattering (DLS) size distribution of P/B-COS NPs. (c) Fourier transform infrared spectroscopy (FTIR) of PLGA, COS, and P/B-COS NPs. (d) Hemolysis ratio (%) of red blood cells cultured with P/B NPs at various concentrations. A ratio less than 5% was regarded as no obvious hemolysis (means ± SD, *n* = 3). (e) The stability of PLGA NPs, P/B NPs, and P/B-COS NPs in phosphate-buffered saline (PBS) during 48 h were characterized by TSI value. (f) Cumulative BUD release of P/B NPs and P/B-COS NPs in PBS at 37°C and 60°C. ^∗^ indicates *p* < 0.05, ^∗∗^ indicates *p* < 0.01, ^∗∗∗^ indicates *p* < 0.001.

We characterized the morphology of nanoparticles by scanning electron microscope. The particle structure was well preserved but some adhesion between each particle was observed; it may be caused by encapsulated chitosan oligosaccharides (Figure 2(a) and Figure S2). Dynamic light scattering (DLS) detection revealed the P/B NPs had a mean hydraulic diameter of ≈153 nm with a narrow size distribution (PDI = 0.095). After coating the nanoparticles with COS, the average diameter becomes larger, up to 196 nm with a wide size distribution (PDI = 0.213) (Figure 2(b)). The introduction of COS greatly changed the zeta potential from -10.9 mV to 0.812 mV. PLGA nanoparticles possessed a mean zeta potential of −10.9 ± 0.91 mV, which was caused by the carboxyl group on the PLGA NPs. P/B-COS NPs had a mean zeta potential of 0.812 ± 0.06 mV due to COS addition. The BUD-loading capacity and encapsulation efficiency were 8.34% and 83.4%, respectively (Table S1). Component analysis of PLGA NPs and P-COS nanoparticles was performed by FTIR; the peaks were comparatively analyzed as shown in Figure 2(c). A strong peak at 1760 cm^−1^ corresponding to the carbonyl stretching frequency can be observed in both the PLGA and synthetic product P-COS NPs.

The peaks at 890 cm^−1^ and 1402 cm^−1^ were identified as characteristic absorption peaks of *β*-pyran glycosidic bonds and carbon nitrogen bond in both the chitosan and the product. The amide I band in 1654 cm^−1^ is due to the stretching vibrations of the carbon oxygen double bond in the product. Thus, these results indicated that COS was connected to the surface of PLGA NPs successfully.

A hemolysis assay was conducted since nanoparticles need to be administered intravascularly. The result showed that NPs had only a slight influence on blood erythrocytes, and the hemolysis ratio was consistently lower than 5% within the concentration range of 2.5–500 *μ*g/mL (Figure 2(d)). The interactions between NPs and blood components could change the size and the stability of particles. The stability of NPs was evaluated by the parameter of Turbiscan stability index (TSI, ranges from 0 to 100). The smaller TSI value indicated the better stability of the suspension. It showed that the NPs remained stable in 10% FBS within 72 h. When COS was grafted on the surface of nanoparticles, the stability decreased slightly indicating that it is easier to aggregate in DMEM (Figure 2(e) and Figure S3).

To investigate the drug release behavior of NPs, an in vitro drug release assay was conducted. The PLGA NPs showed an initial burst release followed by a slow drug release at 37°C, which indicated that these NPs could remain stable under physiological conditions. After 8 h, P/B-COS NPs released about 20% BUD under 37°C, and this ratio increased to about 79.6% under 60°C (Figure 2(f)). In order to characterize the drug release more intuitively, we detected the morphological changes of nanoparticles through the short-term heating experiment. According to the results from the SEM picture, it can be found that the nanoparticles was ruptured after being placed at 60°C for 1 min, resulting in drug release. This result suggested that the drug release behavior of P/B-COS NPs was temperature-dependent, and this feature may be due to the thermal effect of the ablation; the rapid increased local temperature generated exceeds the Tg of PLGA. It thus leads to the collapse and cracks of the NPs, resulting in release of the BUD.

### 2.2. In Vitro Cytocompatibility and Anti-Inflammation Activities

**Figure 3:**
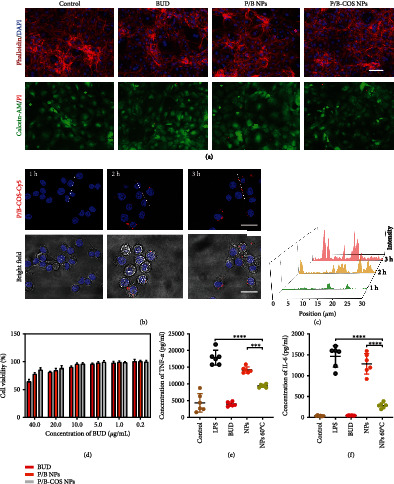
Biocompatibility and effectiveness verification in vitro. (a) Fluorescence images of stained cardiac fibroblasts in the different group. (Cytoskeleton: phalloidin, red fluorescence; cell nucleus: DAPI, blue fluorescence; live cells: calcein-AM, green fluorescence; dead cells: PI, red fluorescence, scale bar = 100 *μ*m). (b) The intracellular uptake of P/B-COS-Cy5 NPs by RAW264.7 cells after incubation with the different times and detected by confocal laser scanning microscope (cell nucleus: Hoechst, blue fluorescence; P/B-COS-Cy5 NPs: ed fluorescence, scale bar = 20 *μ*m). (c) Fluorescence quantitative analysis of the intracellular uptake of NPs in (b). (d) The cytotoxicity evaluation of NPs using CCK-8 assay in cardiac fibroblasts with different concentration (means ± SD, *n* = 3). (e) TNF-*α* and (f) IL-6 concentration in culture supernatant. Evaluation of anti-inflammatory effect of NPs in RAW264.7 cell (means ± SD, *n* = 6, ^∗∗∗^ indicates *p* < 0.001, ^∗∗∗∗^ indicates *p* < 0.0001).

We explored the cytocompatibility of the materials to myocardial fibroblasts and the anti-inflammatory activity of BUD released from the P/B-COS NPs with RAW 267.4 cells in vitro. Fluorescence images (cytoskeleton and cell nucleus, live and dead cells) of stained myocardial fibroblasts cells in the different groups were shown in Figure 3(a). There was no significant difference between the groups. The viability of the cells was above 85% when the BUD is in 10 *μ*g/mL (Figure 3(d) and Figure S4), and the concentration of the proinflammatory mediators (IL-6 and TNF-*α*) induced by lipopolysaccharide (LPS) was obviously decreased after treatment with BUD, there was no significant difference compared with the control group. Compared with LPS group, for NPs group, the concentration of TNF-*α* and IL-6 was decreased to 79.5% and 87.9%; the anti-inflammatory effect may be caused by surface adsorption and release of drugs in the early stage and the internalization of NPs by RAW264.7 and following intracellular drug release. As shown in Figure 3(b), with the extension of incubation time, the intracellular uptake of drugs gradually increased and showed stronger red fluorescence. The quantitative analysis by Image J also verified this conclusion (Figure 3(c)). For NPs 60°C group, the concentration of TNF-*α* and IL-6 was decreased to 52.8% and 19.4% of the LPS group, respectively; the anti-inflammatory effect was significantly higher than that of NPs group, indicating the increase of drug release caused by short-term high temperature (Figures 3(e) and 3(f)). These results suggested that the NPs had good biocompatibility and the drugs retain their bioactivity during the release.

### 2.3. Pharmacodynamics and Preliminary Safety of P/B-COS NPs in Mice

**Figure 4:**
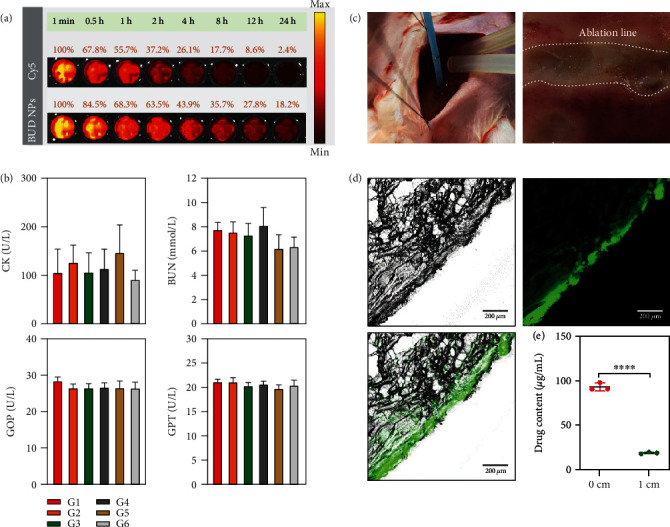
Drug release profile and preliminary safety evaluation. (a) The circulation profile of Cy5-labeled P/B-COS NPs in the blood. Blood was extracted at different time points after the NPs were injected into the tail vein of C57BL/6 mice and imaged under a fluorescence imaging system. The fluorescence intensity at one minute was normalized to be 100%. (b) Serum biochemistry data of mice treated with PBS (G1), free BUD (G2), PLGA NPs (G3), P/B NPs (G4), P-COS NPs (G5), and P/B-COS NPs (G6) (at an equivalent dose of 1 mg/kg BUD), respectively (*n* = 4, means ± SD). (c) Photos of ablation on rabbit thigh muscle, the white dotted line is a continuous ablation line. (d) P-COS nanoparticles entering the ablation site of rabbits were characterized by fluorescein isothiocyanate (FITC). (e) The concentration of budesonide was calculated by HPLC on and away from the line (^∗∗∗∗^ indicates *p* < 0.0001).

The circulation profile of the NPs was investigated using the Cy5 probe. The result showed that the fluorescence signal of P/B-COS NPs was significantly stronger than that of free Cy5 at different times. The fluorescence signal of P/B-COS NPs in the blood remained relatively higher (18.2%) at 24 h (Figure 4(a)), and over half of P/B-COS NPs were cleared at 4 h. The results suggested that the NPs could prolong the lifetime and a rapid clearance rate. Biochemical analysis and blood routine examination of the serum was also conducted to evaluate organ toxicity. The levels of CK (creatine kinase), BUN, GOP, and GPT indicated that all groups could not cause liver injury and kidney injury (Figure 4(b)). There were no significant changes in the concentrations of leukocytes, erythrocytes, and platelets (Figure S5). The preliminary safety evaluation demonstrated that NPs were biocompatible and had clinical transformation potential.

### 2.4. P/B-COS NPs Delivered into the Localized Muscle Tissue of Rabbits

Four ablation lines containing 40 points were made on P/B-COS NPs irrigation and saline irrigation group each. Ablation produced an obvious lesion on gracilis muscle. Contact force (11.63 ± 2.23 vs. 10.57 ± 1.56, *p* = 0.495) and impedance (82.03 ± 7.60 vs. 84.50 ± 5.43, *p* = 0.283) showed no statistically significant difference between groups. The concentration of BUD was higher near the P/B-COS NP irrigation line than that away from the line (*p* < 0.0001) (Figure 4(e)). PLGA-FITC-COS irrigation ablation group was insignificant in contact force (12.17 ± 1.94, *p* = 0.198) and impedance (88.67 ± 5.72, *p* = 0.091) compared with P/B-COS NP irrigation and saline irrigation groups. Fluorescent imagining indicated that P-COS NPs could infiltrate into local tissue via irrigation (Figure 4(d)). PLGA nanoparticles without COS modification can also enter local tissues by ablation (Figure S6).

### 2.5. Ablation Procedures in the Left Atrium

**Figure 5:**
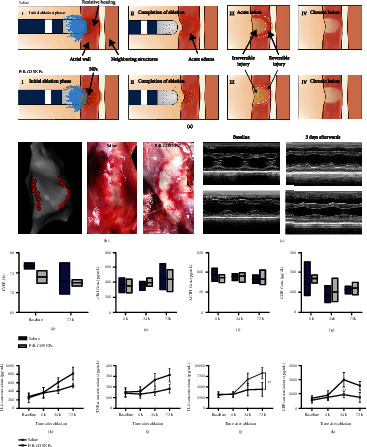
Evaluation of anti-inflammatory effect on pig models. (a) Schematic illustration of localized myocardial anti-inflammatory effects of budesonide during radiofrequency catheter ablation. Reversible lesions and inflammatory responses tend to be milder adjacent to the ablation site. (b) Electroanatomical mapping findings in pigs and the gross pathological results of pigs three days after ablation. (c) M-mode echocardiography in pigs showing the LV wall motion of the heart before and three days after ablation. (d) LVEFs were measured by echocardiography before cardiac ablation (baseline) and 3 days afterward (endpoint). The serum concentration of cTnl (e), ACTH (f), and COR (g) immediately after ablation (0 h) and 24 hours/72 hours later. Serum concentration of IL-6 (h), TNF-*α* (i), IL-10 (j), and CRP (k) at different time after cardiac ablation. All data are presented as means ± SD. *n* = 3 animals per group (^∗^*p* < 0.05).

The whole ablation and lesion formation process can be divided into four phases (Figure 5(a)). During the initial ablation phase, saline is sprayed to the local myocardium through the catheter; the nanoparticles can enter the local myocardial tissue. Due to the resistive heating, the temperature of tissue at 3 mm depths can exceed 60°C after 5 s and continue to rise to more than 90°C, and then, the temperature decreases slowly. The temperature of the whole process is ≥50°C for more than 50 s. Therefore, the nanoparticles can reach the glass transition temperature (45°C), resulting in particle breakage and effective release of drugs [26].

The nanoparticles rupture and release anti-inflammatory drugs. After ablation, the drug in the administration group was continuously released, and the myocardium further formed an area of irreversible injury and an area of reversible injury. The reversible injury area in the normal saline group was larger than P/B-COS NPs group. 72 h later, the photos of myocardial tissue exhibit significantly milder degree of edema and inflammation around the myocardial ablation line in the P/B-COS NP group than that in the saline group (Figure 5(b)).

Six ablation lines (173 points) were made in the LA in each group. No statistically significant differences were shown in the time of ablation (33.29 ± 7.00 vs. 33.54 ± 5.31, *p* = 0.788), contact force (10.12 ± 2.05 vs. 10.17 ± 1.81, *p* = 0.864), and AI (448.75 ± 38.18 vs. 452.31 ± 25.70, *p* = 0.472) (Table S2). Echocardiography indicated that heart function was not affected by the procedure (Figures 5(c) and 5(d)). A similar extension of myocardial necrosis during 72 h was implied by cardiac troponin I (cTnI) (Figure 5(e)). The pituitary function was not disturbed by the perfusion of P/B-COS NPs. There was no difference in ACTH and COR between the two groups during 72 h (Figures 5(f) and 5(g)). Pigs' organs were sliced and stained with HE, and there was no morphological difference between the saline groups and the P/B-COS NPs group after operation (Figure S8).

### 2.6. Anti-Inflammatory Effect of P/B-COS NPs

Systemic inflammation was characterized by inflammatory factors in the blood. The results showed that the concentration of inflammatory factors IL-6, TNF-*α*, IL-8, and CRP and anti-inflammatory factors IL-10 did not change significantly before and immediately after ablation. Inflammatory factors in both groups increased, but 72 hours later, the level of inflammatory factors in the saline group increased more significantly except the concentration of IL-8. The concentration of anti-inflammatory factor IL-10 in NPs group was higher than that in the saline group at 72 h, with significantly different (Figures 5(h)–5(k) and Figure S7). The results showed that P/B-COS NPs had obvious systemic anti-inflammatory effect within three days after administration.

**Figure 6:**
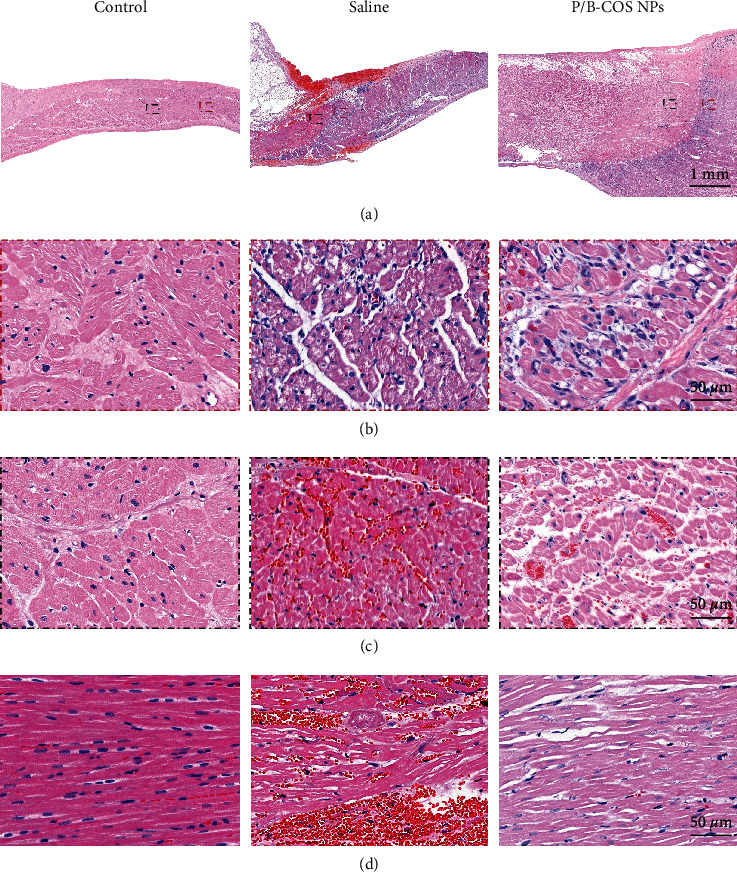
Histological findings of radiofrequency ablation lesions. (a) Hematoxylin and eosin (HE) staining myocardial slices showed local changes, normal myocardium (control), tissue ablation with saline (saline group) and ablation with NPs treatment (P/B-COS NPs), acute bleeding, and tissue injury adjacent to the ablation line. (b) In the enlarged view (red dotted bordered rectangle in (a)) of the local reversible injury site after ablation, there was more obvious inflammatory cell recruitment in the saline group. (c) Transverse (enlarged view of the black dotted bordered rectangle in (a)) and (d) longitudinal sections of myocardium at normal myocardium and ablation site of local irreversible injury. There was significant hematoma in the saline group.

HE-stained sections of myocardial tissue at the ablation site were used to describe local inflammation (Figure 6(a)). Compared with normal myocardium (control group), the boundary between reversible injury and irreversible injury of ablated myocardium was obvious, and the ablation line (white dotted line) was clear. The statistics of tissue sections in each group showed that the hemorrhage area in the saline group was significantly larger than that in the control group, and there was no significant difference between the P/B-COS NPs group and the control group (Figure S9). In the saline group, there was an obvious hematoma near the ablation line; the recruitment of inflammatory cells was more obvious in the enlarged view of the local reversible injury site after ablation (Figure 6(b), the rectangle with the red dotted line in Figure 6(a)). The cross-section (Figure 6(c), enlarged view of black dotted line in Figure 6(a)), and longitudinal section (Figure 6(d)) of the ablation site of normal myocardium (control) and local irreversible injury show that the cardiomyocyte gap in the ablation group was enlarged, and the myocardium underwent coagulative necrosis, and there was no significant difference in the ablation effect. Free BUD can also enter the local myocardium and play a certain anti-inflammatory effect, but the anti-inflammatory effect is not as good as that of the P/B-COS group (Figure S10), which may be because the free drugs are cleared rapidly in the tissue and cannot play a long-term anti-inflammatory effect.

## 3. Discussion

Myocardial inflammation and edema caused by radiofrequency energy have been implicated in early recurrence. The study from Matthew applied near field ultrasound to reveal that ablation energy can trigger tissue edema formation immediately and increased wall thickness by 25% in the median. A strong inflammatory activation was initiated after catheter ablation [27]. The inflammatory response indicated by a surge in hs-CRP will intensify within 3 days [28]. The most effective anti-inflammatory treatment as a means of reducing early recurrences after AF ablation is by applying glucocorticoids. Many ways of administering GCs have been reported in several researches. Takashi Koyama gave intravenous hydrocortisone (2 mg/kg) to patients the day of the procedure, and oral prednisolone (0.5 mg/kg/day) was administered for 3 days after the PVI [10]. Kim et al. used an intravenous bolus of 0.5 mg/kg of methylprednisolone for 2 days followed by 12 mg daily dosage of oral methylprednisolone for 4 days [29]. In both studies, a sequential preprocedural intravenous dose and postprocedural oral doses were used. However, it is difficult for the drug to reach the local ablation site and play a role, which mainly produces systemic anti-inflammatory effect. A single high-dose steroid therapy does not change the level of CRP and is not sufficient to prevent early postoperative recurrence of atrial fibrillation. Thus, the prophylactic corticoid administered into systemic circulation will not take effect on ablation lesion of local myocardium.

Localized GCs delivering method was used in cardiology as drug-eluting on the pacemaker. Intravenous pacing lead results in acute injury to cellular membranes which leads to myocardial edema. 1 mg of dexamethasone was impregnated at the electrodes' tip. It aimed at coping with an increase in pacing impedance caused by myocardial inflammation, edema, and fibrosis [30, 31]. BUD not only features anti-inflammatory effects like dexamethasone but presents a unique characteristic of high first-pass effect and very quick clearance. So, it is widely used as atomization and oral administration in treating asthma or inflammatory bowel diseases, respectively. The idea of using BUD irrigation was to deliver enough amount of GCs to ablate the myocardium and reduce the systemic effects. We used PLGA as drug carriers, loaded with anti-inflammatory drug budesonide to construct a temperature sensitive nanoparticle. Ablation of local myocardial with high temperature leads to the rupture of nanoparticles and the release of anti-inflammatory drugs to achieve the immediate anti-inflammatory effect. At the same time, some particles enter the local myocardium, and the drugs are slowly released. Compared with free drugs, it plays a long-term anti-inflammatory role with good biocompatibility. Ablation in the left atrium of pigs showed that by adding P/B-COS NPs to irrigation saline, the results of ablation, heart function, and pituitary function did not alter. Anti-inflammatory effect could last up to 72 h after the procedure. Further histological findings suggested a similar necrotic area but milder inflammatory cell infiltration and hemorrhage. The anti-inflammatory mechanisms of P/B-COS NPs could be like other local glucocorticoids. Just like the steroid-eluting electrode which uses dexamethasone, the introduction of BUD decreases the release of oxidant, oxygen-free radicals, hydrolytic enzymes, and other inflammatory mediators and thus decreases inflammatory injury on myocardium near ablation sites [31].

Since there was no study on intravenous BUD, the density of P/B-COS NPs were calculated based on a former experiment on acute lung injury treatment. The irrigation flow rate and ablation power were set to 25 mL/min and 30 W to prevent possible steam pop and meet proper drug density. However, the flow rate in daily practice with STSF catheter was 15 mL/min and ablation power can reach up to 90 W, which means less drug administration in a shorter time. Whether such an amount of BUD can still be presented with anti-inflammatory effects warrants further test. Secondly, due to the complexity of atrial fibrillation model, we only studied the effect of drug administration on local and systemic inflammation in the experiment, whether the anti-inflammatory effect can directly reduce the recurrence of atrial fibrillation needs further clinical study.

The present study provides an ideal way for delivery of glucocorticoid to localized myocardium with saline irrigation during catheter ablation. The newly composited P/B-COS NPs can form a stable solution. It can be safely administered into the bloodstream with a prolonged local anti-inflammatory effect and quick systemic clearance. This administration strategy can achieve efficient drug delivery in the local myocardium, significantly reduce myocardial hematoma, and decrease local and systemic inflammation after ablation without adding additional processes to the procedure. The results of this study not only provide a brand-new medication for anti-inflammation therapy but also open a different applicable method for localized drug administration.

## 4. Materials and Methods

### 4.1. Fabrication of PLGA NPs

The process for the synthesis of P/B-COS NPs was prepared by ultrasonic emulsification and is illustrated in Figure 1(b) and Figure S1. Firstly, BUD (1.5 mg) and PLGA (10 mg) was dissolved in 0.5 mL dichloromethane (CH_2_Cl_2_), then the reaction mixture was dripped to the deionized water containing 2% PVA. Then, the ultrasonic homogenizer was used at 150 W ultrasonic for 5 s and stopped for 3 s for 8 mins. The organic solvent was evaporated at 40°C for 30 mins with a rotary evaporator to obtain the nanodispersion system. Finally, it was centrifuged at 16000 rpm/min for 20 mins and washed with deionized water thrice. After lyophilization, the resulting white nanoparticles were stored at -20°C for further experiments.

### 4.2. Preparation of P/B-COS NPs

100 mg of prepared nanoparticles were dispersed in 5 mL MES buffer containing 0.1 M NHS and EDC, activated for 3 h, then 2 mg/mL of prepared chitosan solution was added to the above reaction system, and stirred at room temperature for 24 h, centrifuged at 16000 rpm/min, and freeze-dried to obtain the product.

### 4.3. Encapsulation Efficiency and Physical Characterization

The concentration of BUD was determined by reversed-phase high-performance liquid chromatography (RP-HPLC Agilent 1100, CA). Briefly, 10 mg of lyophilized particles were stirred in 10 mL of ethyl acetate for 4 hours. Then, the ethyl acetate residue was completely volatilized, dissolved in 5 mL acetonitrile phosphoric acid buffer with a pH 3 (4 : 6) for 2 h, then filtered with 0.45 *μ*m, and the filtrate was analyzed by reversed-phase high-performance liquid chromatography (RP-HPLC Agilent 1100, CA). Chromatographic conditions were as follows, chromatographic column: Purospher STAR LP, RP-18 endcapped, (Merck, Germany) 5 *μ*m; flow rate: 1.2 mL/min; eluent: phosphate buffer (pH 3): acetonitrile = 40 : 60, injection volume: 20 *μ*L. The calculation of drug loading efficiency and encapsulation efficiency refers to the previous literature [32].

### 4.4. Hemolysis Assay In Vitro

We performed a hemolysis assay to study the interaction of different nanoparticles with blood erythrocytes. 2% RBC suspension from C57BL/6 mice was incubated with different concentrations of BUD for predetermined periods at 75 rpm and 37°C. The RBC suspension treated with PBS and deionized water was used as a negative and positive control, respectively. Then, each sample was centrifuged at 1000 rpm for 5 mins at each time point. The absorbance of the supernatant was measured at 577 nm on 96 well plates; 655 nm absorbance was used as reference. The percentage of hemolysis is calculated according to the following formula:

(1) $$\begin{array}{c} \text{Hemolysis ratio }\left(\%\right)=\frac{\left(A_{\text{sample}}-A_{0\%}\right)}{\left(A_{100\%}-A_{0\%}\right)}\times 100\%. \end{array}$$

### 4.5. Stability of the Nanoparticle

Turbiscan vertical scan analyzer (formulation SA, France) was used to measure the light scattering. 0.2 mg/mL PLGA NPs, P/B NPs, and P/B-COS NPs were dispersed in DMEM with 10% FBS at room temperature. The curve of transmission percentage (T%) with height is obtained by the instrument for a fixed time [33].

### 4.6. In Vitro Anti-Inflammatory Effect

To evaluate the bioactivity of BUD released from the P/B-COS NPs, RAW264.7 cells were next plated in 24-well plates at a density of 1.0 × 10^4^ cells/well and cultured in DMEM containing 10% fetal bovine serum and 1% penicillin-streptomycin at 37°C with 5% CO_2_. The cells in the control group were not treated, the solution containing the same amount of BUD (10 *μ*g/mL) was added to the BUD, NPs (P/B-COS NPs), and NPs 60°C (NPs were treated at 60°C for 3 mins before being added to the culture medium) groups, respectively, followed by stimulated with 1 *μ*g/mL LPS for 24 h. The content of TNF-а and IL-6 in cell culture medium was quantified by ELISA kits.

To visualize the cellular phagocytosis of drugs, Cy5-labeled NPs was added to RAW264.7 cells in confocal dish. After incubation for different times, remove the medium. Then, the cells that had swallowed the NPs were washed gently with PBS twice followed by the nuclear fluorescent dye Hoechst 33342 added to the dishes for 10 min to dye the cell nucleus. Finally, the fluorescent dye that was not bound to the nucleus was washed away with PBS, and the cells were observed by confocal laser scanning microscope (CLSM).

### 4.7. Measurement of Drug Release with Ablation on Rabbit Thigh Muscle

Four New Zealand white rabbits (3-3.5 kg) were used for the experiments. General anesthesia was achieved with intravenous phenobarbital and maintained with isoflurane inhalation. A 5 cm incision was made over the skin of the right thigh, exposing the muscles, freeing the subcutaneous tissue, and lifting it to form a cradle. Linear ablation line (white dotted line) was made on the thigh muscle (Figure 4(c)). 5000 units of heparin were added to 400 mL saline and circulated through an external pump at 37°C at an exchange flow rate of 200 mL/min. Thigh muscle electric models were mapped under the guidance of CARTO 3 system. The ablation catheter (56-pore Thermocool SmartTouch SF Catheter, Biosense Webster) was positioned perpendicular to the tissue, with pressure around 10 g against the muscle. The ablation time was set to 60 s, power was 30 W. With the BUD concentration is 8 mg/500 mL, a flow rate of 30 mL/min will deliver BUD at 480 *μ*g/min. The rabbits were euthanized immediately after the ablation, and gracilis muscle was collected. Linear ablation lesions were sliced on transverse histological sections. The concentration of BUD was calculated on and 1 cm away from the line.

### 4.8. Localized Drug Delivery on Ablation of Pig Atria

This study included a total of 6 Bama miniature pigs weighing 38 to 42 kg. The animals received an intravenous injection of 4 mg/kg propofol for general anesthesia. Under electrocardiographic monitoring and pulse oximetry, the animals were placed in the supine position, intubated, and maintained on artificial ventilation. Under sterile surgical conditions, a 10-polar catheter (APTMedical, Triguy, China) was placed in the coronary sinus through the internal jugular vein under fluoroscopic guidance. Transseptal puncture with 8.5 F long sheath (SL1-St. Jude Medical Inc.) was performed via the right femoral vein for access of the left atrium.

High density electroanatomical mapping of atrial chambers was performed with PentaRay NAV catheter (BiosenseWebster, USA). Supplemental Movie 1 demonstrates the mapping process. The cyan mesh and grey mesh represent the left atrium and the right atrium, respectively. Linear ablations were carried out using 56-pore Thermocool SmartTouch SF Catheter (Biosense Webster, USA) under CARTO 3 guidance. The irrigation flow rate was 30 mL/min when ablation started and 2 mL/min when ablation stopped. Supplemental Movie 2 demonstrates real-time visualization of in vivo saline irrigation using intracardiac ultrasound. Three pigs received irrigation of saline as the control group (CG) and the other three received saline with P/B-COS NPs (16 *μ*g/mL) solution. One pig received normal saline containing free BUD as a parallel control (same dosage of BUD). Ablation power was set to 30 W, with a contact force at around 10 g. The target ablation index (AI) was 450. Two point-by-point ablation lines were made in the posterior wall of the left atrium. Supplemental Movie 3 demonstrates ablation on the posterior wall of a pig's left atrium. After the operation, the catheter was removed, and the wound was sutured. All animals received rivaroxaban 10 mg after ablation for anticoagulation and gentamicin 80 mg for infection prophylaxis.

### 4.9. Inflammatory Characterization after Cardiac Ablation

Blood samples were collected after intubation of femoral veins on day one and day three before sacrifice, and centrifuged immediately. Troponin, COR, ACTH, IL-6, IL-8, IL10, TNF-*α*, and CRP were determined at each time point with proper ELISA kits.

The animals were sacrificed three days after the procedure with a lethal KCl injection under general anesthesia. The hearts were carefully removed, and the ablation lines were macroscopically identified, correlated with the applications to the cardiac cavities. Special attention was paid to the redness of edema for macroscopic analysis. Linear ablation lesions were sliced on transverse histological sections. The slides were stained with hematoxylin-eosin (HE) for analysis of hemorrhage and inflammatory response.

### 4.10. Animal Model Experiment

The experimental process was strictly in line with the “Beijing Administration Rule of Laboratory Animal” and the Institutional Animal Care and Use Committee (IACUC) approved protocol of the Animal Care Center at the Capital Medical University.

### 4.11. Statistical Analysis

Statistical analyses were performed using Origin software program and GraphPad Prism v.7.0. The continuous variables were analyzed descriptively as mean ± SD; Student's *t* test or one-way ANOVA was used to compare the difference between groups. *p* value of 5% or less was statistically significant.

## Supplementary Materials

Supplementary Materials Text ST1: materials. Text ST2: characterization of NPs. Text ST3: drug release profile. Text ST4: circulation profile of Cy5-labeled NPs. Text ST5: drug release profile. Text ST6: preliminary safety evaluation. Figure S1: schematic diagram of nanoparticle preparation process. Figure S2: dynamic light scattering (DLS) size distribution of P/B NPs. Figure S3: transmittance (T%) profile of different nanoparticle solutions. Figure S4: the cytotoxicity evaluation of NPs. Figure S5: blood routine examination of mice. Figure S6: PLGA nanoparticles entering the ablation site of rabbits. Figure S7: cytokine measurement at different stages of ablation. Figure S8: H&E staining assays of major organs collected from pigs on the third day after cardiac ablation. Figure S9: statistics of hemorrhage area in tissue sections in each group. Figure S10: histological findings of radiofrequency ablation lesions in free BUD group. Table S1: characteristics of P/B and P/B-COS NPs size and zeta potential of nanoparticles according to the chitosan coating and inclusion of BUD. Table S2: ablation parameters of control group (CG) administration of physiological saline in comparison with the experiment group (EG) administration of P/B-COS NPs. Movies S1: the mapping process before radiofrequency catheter ablation. Movie S2: real-time visualization of in vivo saline irrigation using intracardiac ultrasound. Movies S3: real time monitoring of left atrial posterior wall ablation in pigs.
