# Supplementary material for: Localized Myocardial Anti-Inflammatory Effects of Temperature-Sensitive Budesonide Nanoparticles during Radiofrequency Catheter Ablation
Source: Research (Wash D C). 2022 May 31;2022:9816234. doi: 10.34133/2022/9816234 (PMC9178488; doi:10.34133/2022/9816234)
Supplement: Supplementary Materials — Text ST1: materials. Text ST2: characterization of NPs. Text ST3: drug release profile. Text ST4: circulation profile of Cy5-labeled NPs. Text ST5: drug release profile. Text ST6: preliminary safety evaluation. Figure S1: schematic diagram of nanoparticle preparation process. Figure S2: dynamic light scattering (DLS) size distribution of P/B NPs. Figure S3: transmittance (T%) profile of different nanoparticle solutions. Figure S4: the cytotoxicity evaluation of NPs. Figure S5: blood routine examination of mice. Figure S6: PLGA nanoparticles entering the ablation site of rabbits. Figure S7: cytokine measurement at different stages of ablation. Figure S8: H&E staining assays of major organs collected from pigs on the third day after cardiac ablation. Figure S9: statistics of hemorrhage area in tissue sections in each group. Figure S10: histological findings of radiofrequency ablation lesions in free BUD group. Table S1: characteristics of P/B and P/B-COS NPs size and zeta potential of nanoparticles according to the chitosan coating and inclusion of BUD. Table S2: ablation parameters of control group (CG) administration of physiological saline in comparison with the experiment group (EG) administration of P/B-COS NPs. Movies S1: the mapping process before radiofrequency catheter ablation. Movie S2: real-time visualization of in vivo saline irrigation using intracardiac ultrasound. Movies S3: real time monitoring of left atrial posterior wall ablation in pigs. [file 9816234.f1.zip › Liu_Revised-Supplementary_Material.docx]

Supplementary Materials for

Localized Myocardial Anti-inflammatory Effects of Temperature Sensitive Budesonide Nanoparticles During Radiofrequency Catheter Ablation

Ye Liu^1^†, Lingling Xu^2,3^†, Qiuyun Zhang^4^, Yong Kang^5^, Lifeng Liu^1^, Zheng Liu^1^, Yuxing Wang^1^, Xuejiao Jiang^5^, Yizhu Shan^2,3^, Ruizeng Luo^2,3^, Xi Cui^2,3^, Yuan Yang^2,3^, Xinchun Yang^1^, Xiaoqing Liu^1^**, Zhou Li^2,3,6,7^*

^1^Heart Center & Beijing Key Laboratory of Hypertension, Beijing Chaoyang Hospital, Capital Medical University, Beijing 100020, China

^2^Beijing Key Laboratory of Micro-nano Energy and Sensor, Beijing Institute of Nanoenergy and Nanosystems, Chinese Academy of Sciences, Beijing 101400, China

^3^School of Nanoscience and Technology, University of Chinese Academy of Sciences, Beijing 100049, China

^4^School of Traditional Chinese Medicine, Capital Medical University, Beijing 100069, China.

^5^Academy of Medical Engineering and Translational Medicine, Medical College, Tianjin University, Tianjin 300072, China.

^6^Center on Nanoenergy Research, School of Physical Science and Technology, Guangxi University, Nanning 530004, China.

^7^Institute for Stem Cell and Regeneration, Chinese Academy of Sciences, Beijing 100101, China.

Correspondence should be addressed to Zhou Li; [zli@binn.cas.cn](mailto:zli@binn.cas.cn) and Xiaoqing Liu; [drliuxq@163.com](mailto:drliuxq@163.com)

Ye Liu and Lingling Xu contributed equally to this work.

Content of Supplementary Materials

Text ST1. Materials.

Text ST2. Characterization of NPs.

Text ST3. Drug release profile.

Text ST4. Circulation Profile of Cy5-labeled NPs.

Text ST5. In Vitro Cytotoxicity Analysis.

Text ST6. Preliminary Safety Evaluation.

Figure S1. Schematic diagram of nanoparticle preparation process.

Figure S2. Dynamic light scattering (DLS) size distribution of P/B NPs.

Figure S3. Transmittance (T%) profile of Different nanoparticle solutions.

Figure S4. The cytotoxicity evaluation of NPs.

Figure S5. Blood routine examination of mice.

Figure S6. PLGA nanoparticles entering the ablation site of rabbits.

Figure S7. Cytokine measurement at different stages of ablation.

Figure S8. H&E staining assays of major organs collected from pigs on the third day after cardiac ablation.

Figure S9. Statistics of hemorrhage area in tissue sections in each group.

Figure S10. Histological findings of radiofrequency ablation lesions in free BUD group.

Table S1. Characteristics of P/B and P/B-COS NPs Size and zeta potential of nanoparticles

according to the chitosan coating and inclusion of BUD.

Table S2. Ablation parameters of control group (CG) administration of physiological saline in comparison with the experiment group (EG) administration of P/B-COS NPs

Movies S1. The mapping process before Radiofrequency catheter ablation.

Movie S2. Real-time visualization of in vivo saline irrigation using intracardiac ultrasound.

Movies S3. Real time monitoring of left atrial posterior wall ablation in pigs.

**Supplementary Information Text**

**Text ST1. Materials**

Budesonide (Selleck), poly (lactic-co-glycolic acid) (PLGA) (lactide:glycolide=50:50, ester terminated, molecular weight:40kDa), Chitosan (molecular weight:800-1000; degree of deacetylation:≥93%) and 1-[3-(dimethylamino) propyl]-3-ethyl carbodiimide hydrochloride (EDC) (99%) were obtained from Meilunbio. Polyvinyl alcohol low-viscosity (PVA) (Aladdin). N-hydroxy-succinimide (NHS) (> 98%, Thermo). 2-Morpholinoethanesulfonic Acid (MES) buffer (pH 5.0, Leagene). Cell counting kit-8 (CCK-8) was purchased from Dojindo Laboratories. 4,6-diamidino-2-phenylindole (DAPI), Live/Dead Staining Kit, TNF-а and IL-6 ELISA kits were purchased from Solarbio. Dulbecco’s modified Eagle’s medium (DMEM, Hyclone), fetal bovine serum (FBS, Gibco). All other chemical products were analytical grade and commercially available.

**Text ST2.Characterization of NPs**

The size distribution and surface charge of NPs were characterized by Zetasizer Nano ZS (Malvern, UK). The infrared spectrums were measured by Fourier transform infrared spectrophotometry (FIIR, iS50 ABX, USA). To observe the effect of high temperature on the morphology of nanoparticles, 0.2 mg/mL nanoparticles were dropped on aluminum foil heated to 60℃, dried at room temperature, and characterized by field-emission scanning electron microscopy (SEM, SU8020, HITACHI).

**Text ST3. Drug release profile**

In vitro BUD release behavior of NPs was evaluated. 1 mL nanoparticle dispersions (at an equivalent dose of BUD) were loaded into dialysis bags (7 kDa molecular weight cutoff). Each dialysis bag was immersed in PBS (50 mL, pH 7.4) and incubated in a shaking bed at 75 rpm and 37°C or 60℃. Aliquots (200 μL) of the release media were collected at predetermined time points (0, 2, 6, 12, 24 and 48 h) while the same volume of fresh medium was replenished. The concentration of the released drug was determined by RP-HPLC. To investigated the effect of temperature on the morphology of nanoparticles through short-term heating experiment. The P/B-COS NPs were dropped on the tin foil and kept it at 60℃ for 1 min, and then immediately recovered to room temperature. The rupture of PLGA NPs under 60℃ were characterized by SEM.

**Text ST4. Circulation Profile of** **Cy5-labeled NPs**

To visualize the blood retention time of P/B-COS in vivo, near-infrared fluorescence (NIRF) dye Cy5 was loaded in P/B-COS NPs for time-dependent imaging. 30 μg of free Cy5 and Cy5 labeled P/B-COS NPs were administered to C57BL/6 mice (female, 5 weeks old, 18–22 g) via tail vein injection. Approximately 60 μL of blood was collected in heparin-treated tubes at each predetermined time interval (1 min, 0.5, 1, 2, 4, 8, 24 h), 40 μL of blood from each sample was transferred into a 96-well plate. The dynamic of fluorescence distribution was illustrated by a non-invasive Near Infrared Fluorescence Imaging system. The fluorescence intensity of each group was normalized by considering that the intensity at 1 min was 100%.

**Text ST5. In Vitro Cytotoxicity Analysis**

The cytotoxicity of different formulations was evaluated in primary rat cardiac fibroblasts cells (CFs) by the CCK-8 assay and live/dead cell staining assay. Briefly, CFs were seeded in 96-well plates (3 × 10^3^ cells/well) and incubated for 12 h at 37°C and in a 5% CO_2_ atmosphere. The culture medium was removed, and then, free BUD, PLGA NPs, P-COS NPs, P/B NPs or P/B -COS NPs were added at a series of concentrations and incubated with the cells for an additional 48 h. Then, 10 μL of CCK-8 solution was added to each well, and the plates were incubated for another 2 h. Finally, the absorbance of each group was measured by a Varioskan Flash multimode reader (Thermo, USA) at 540 nm. The cell viability (%) was calculated by the following formula:

Cell viability (%) = A_test_ - A_medium_ / A_control_ - A_medium_ × 100.

Calcein-AM and propidium iodide (Invitrogen USA) were used for live/dead staining. The cytoskeletons and nuclei of the cells were stained with phalloidin and 4′,6-diamidino-2-phenylindole (DAPI), respectively (based on the BUD concentration of 10 μg/mL).

**Text ST6. Preliminary Safety Evaluation.**

To evaluate the preliminary in vivo toxicities of different preparations, healthy male C57BL/6 mice (approximately 5 weeks old, 18-22 g, specific pathogen free (SPF)) were treated with PBS, free BUD, PLGA, P-COS, P/B, or P/B-COS NPs (at an equivalent dose of 1 mg/kg BUD) individually. One week after the administration, whole blood was obtained in anticoagulative tubes for routine blood examination, and serum was obtained for the biochemical analysis.


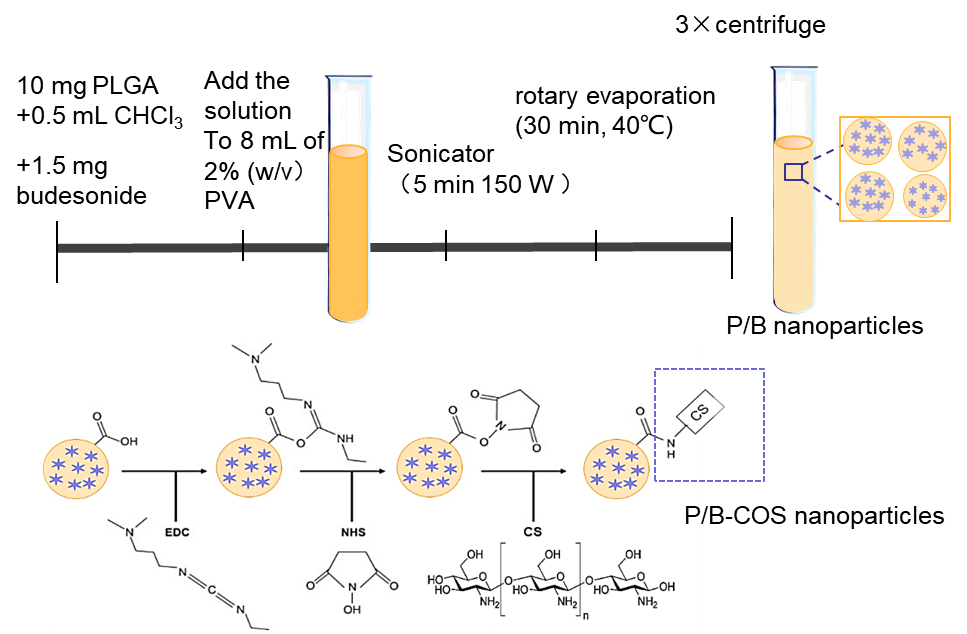


**Figure S1.** **Schematic diagram of nanoparticle preparation process.**


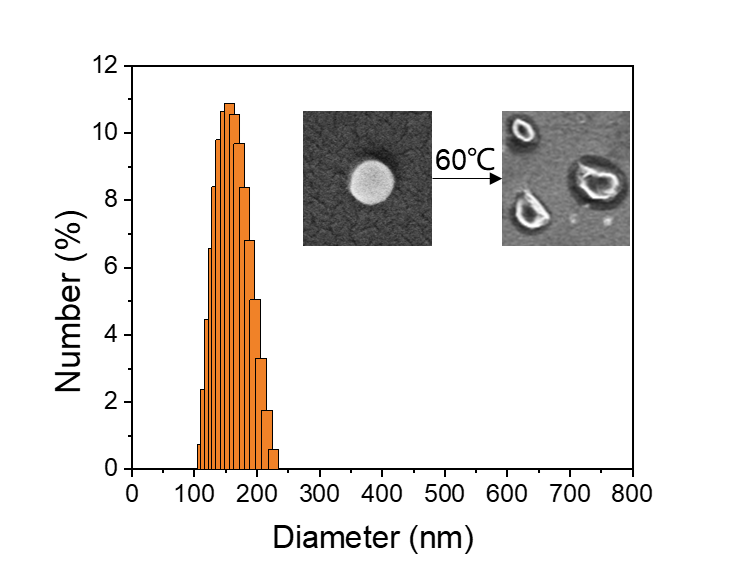


**Figure S2. Dynamic light scattering (DLS) size distribution of P/B NPs.** The rupture of PLGA NPs under 60℃ were characterized by SEM.


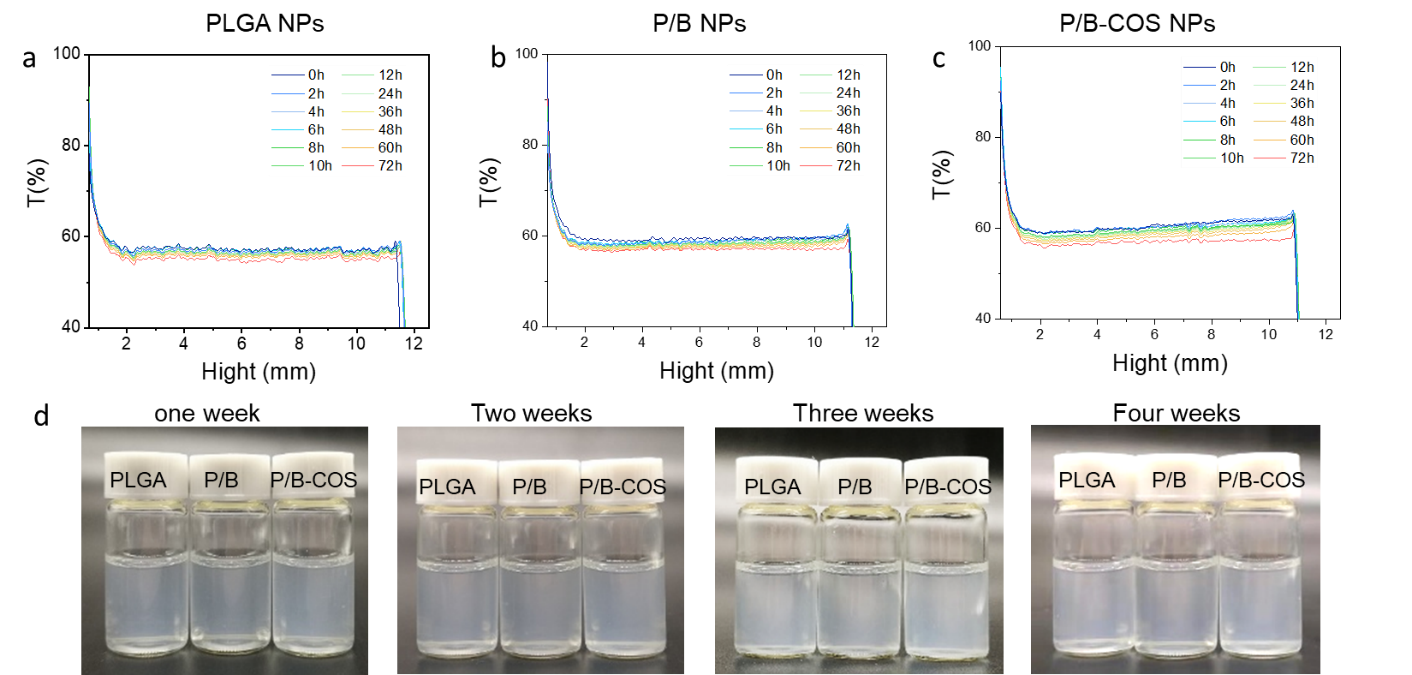


**Figure S3.** **Transmittance (T%) profile of Different nanoparticle solutions.** PLGA NPs (a), P/B NPs (b) and P/B-COS NPs(c) dispersed in the medium as a function of time and tube length. (d) The optical photos of different nanoparticles in PBS.


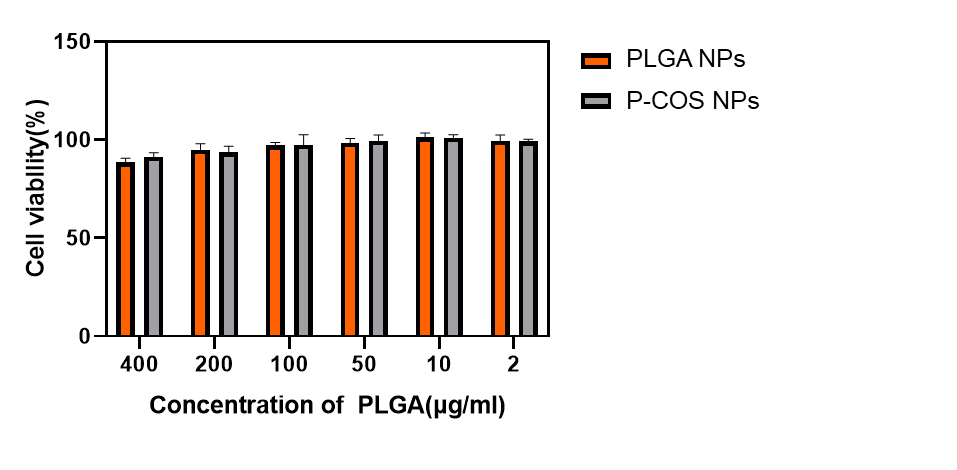


**Figure S4. The cytotoxicity evaluation of NPs.** CCK-8 assay in cardiac fibroblasts with different PLGA concentration.


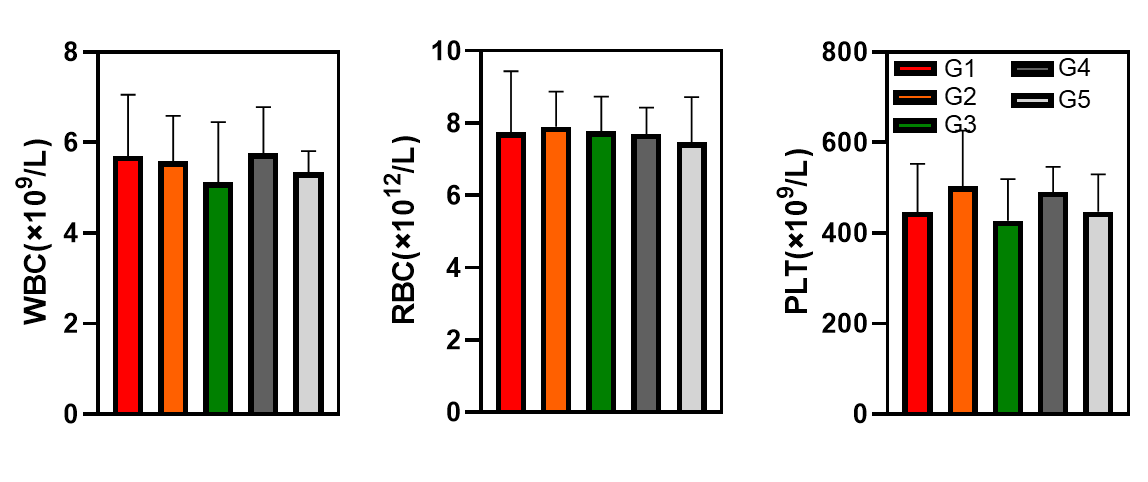


**Figure S5. Blood routine examination of mice.** The mice were treated with PBS (G1), free BUD (G2), PLGA NPs(G3), P/B NPs (G4), P/B-COS NPs (G5).


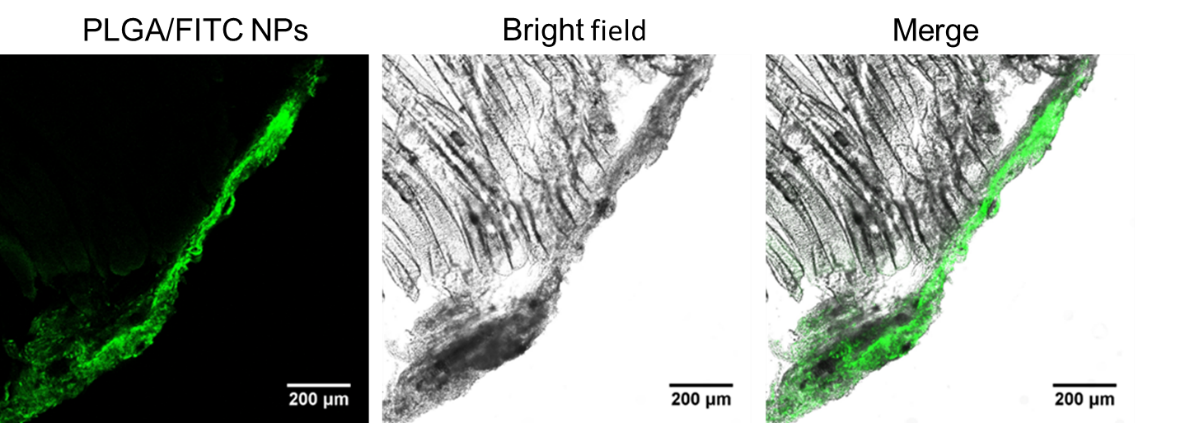


**Figure S6.** **PLGA nanoparticles entering the ablation site of rabbits.** Fluorescent imagining indicated that PLGA NPs could infiltrate into local tissue via irrigation.


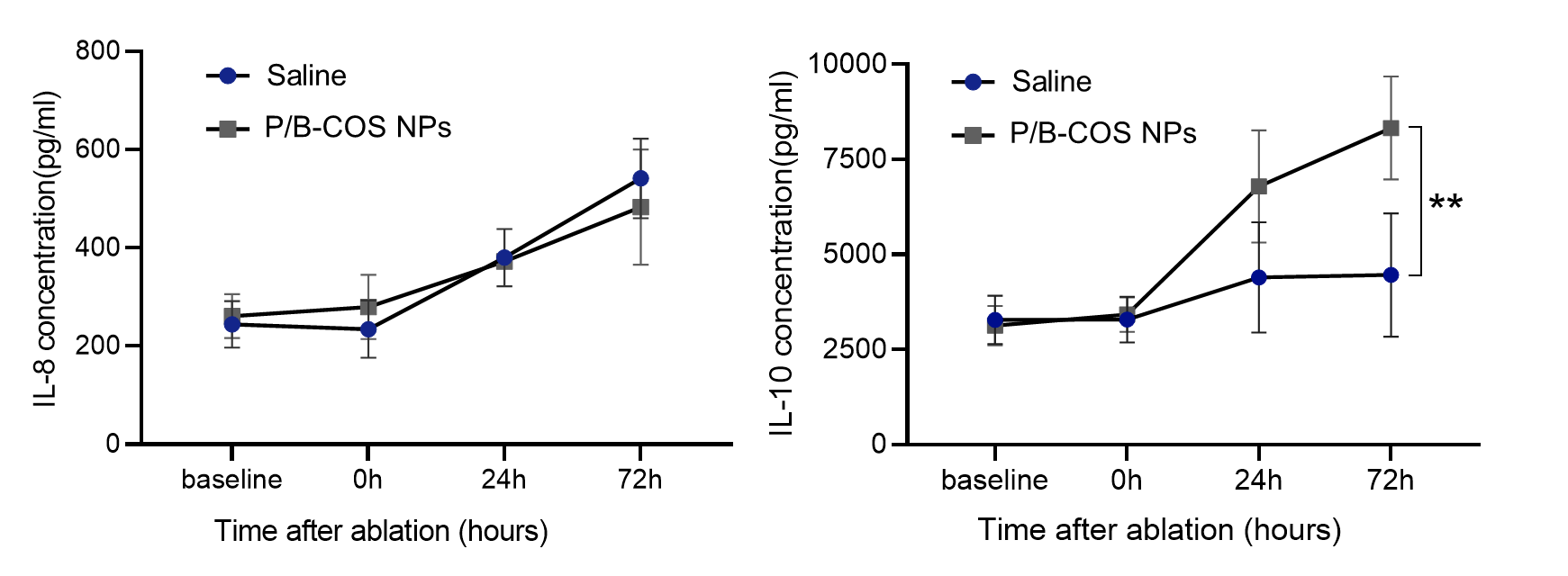


**Figure S7. Cytokine measurement at different stages of ablation.** The concentration of IL-8 (a) and IL-10 (b) in serum at different stages of ablation


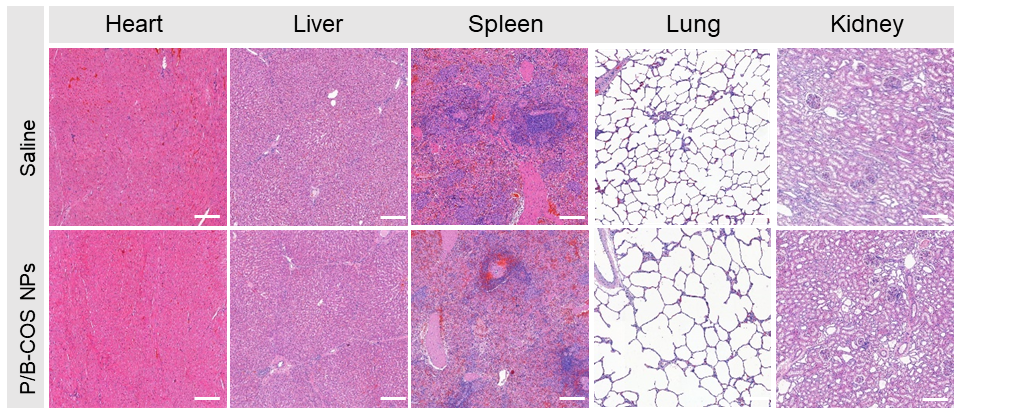


**Figure S8. H&E staining assays of major organs collected from pigs on the third day after cardiac ablation.** (Scale bar 200 μm)

**Fig. S8. Statistics of hemorrhage area in tissue sections in each group.**

**Figure. S9. Statistics of hemorrhage area in tissue sections in each group.**

**
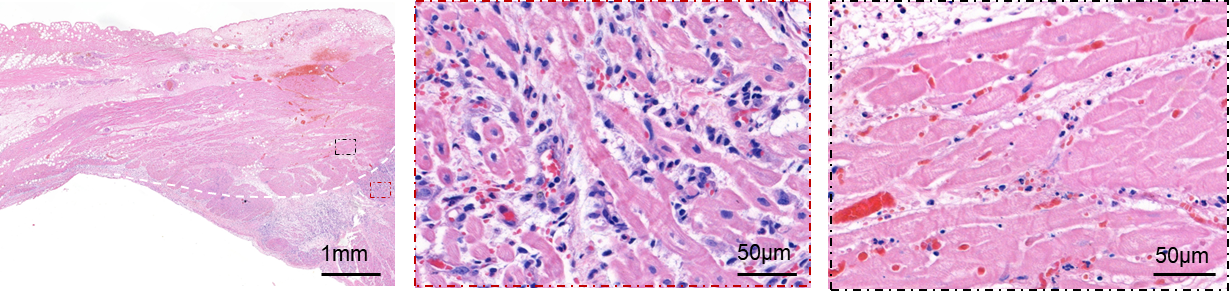
**

**Figure S10.** **Histological findings of radiofrequency ablation lesions in free BUD group.**

**Table S1.** Characteristics of P/B and P/B-COS NPs Size and zeta potential of nanoparticles

according to the chitosan coating and inclusion of BUD. The values are shown as the means ± standard deviation (n=3).

| **Sample** | **Diameter (nm)** | **PDI** | **Zeta potential** | **Loading capacity (%)** | **Encapsulation**  **efficiency (%)** |
| --- | --- | --- | --- | --- | --- |
| P/B-NPs | 153.93±3.11 | 0.095±0.082 | -10.9±0.91 | 8.34±0.41 | 83.4±4.12 |
| P/B-COS NPs | 196.57±40.47 | 0.213±0.099 | 0.812±0.06 | 7.79±0.49 | 77.9±4.9 |

**Table S2**. Ablation parameters of control group (CG) administration of physiological saline in comparison with the experiment group (EG) administration of P/B-COS NPs

| Group | Site Index | Duration Time (s) | Average Force (g) | AI |
| --- | --- | --- | --- | --- |
| CG1 | 37 | 35.36±4.70 | 9.55±2.19 | 454.06±6.73 |
| CG2 | 27 | 31.23±6.76 | 10.77±1.37 | 445.60±45.24 |
| CG3 | 23 | 33.34±2.79 | 10.47±1.28 | 457.37±1.52 |
| EG1 | 25 | 37.14±5.74 | 9.22±2.54 | 457.12±2.71 |
| EG2 | 30 | 32.17±7.53 | 10.12±1.94 | 443.43±48.19 |
| EG3 | 31 | 31.26±6.33 | 10.84±1.36 | 447.14±42.32 |
